# Know Where You Go: Infestation Dynamics and Potential Distribution of Two Bed Bug Species (Hemiptera: Cimicidae) in Africa

**DOI:** 10.3390/insects16040395

**Published:** 2025-04-09

**Authors:** Dennis M. Mbuta, Bonoukpoè M. Sokame, Fathiya M. Khamis, Komivi S. Akutse

**Affiliations:** 1 International Centre of Insect Physiology and Ecology, Nairobi P.O. Box 30772-00100, Kenya; mutuadennis2016@gmail.com (D.M.M.); bsokame@icipe.org (B.M.S.); fkhamis@icipe.org (F.M.K.); 2 Department of Biochemistry, School of Biomedical Sciences, Jomo Kenyatta University of Agriculture and Technology, Nairobi P.O. Box 62000-00200, Kenya; 3 Department of Zoology and Entomology, University of Pretoria, Private Bag X20, Hatfield 0028, South Africa; 4 Unit for Environmental Sciences and Management, North-West University, Potchefstroom 2520, South Africa

**Keywords:** bed bugs, infestation dynamics, distribution, overlap, MaxEnt, suitability zones

## Abstract

Bed bugs (Hemiptera: Cimicidae) are blood-feeding insects closely linked with humans and animals, causing discomfort, and posing potential threats as disease vectors. This study focuses on *Cimex lectularius* (common bed bug) and *Cimex hemipterus* (tropical bed bug), the two key species identified in Africa. *Cimex lectularius* infests human habitats, while *C. hemipterus*, more common in Africa, infests diverse habitats, including bat colonies. In our study, we investigated the infestation dynamics and distribution of bed bugs in Africa, when considering climate, habitat, and host availability using system dynamics and ecological niche modelling techniques. System dynamics modelling analyses in Kenya revealed varied infestation dynamics, with Mombasa having high *C. lectularius* prevalence, Nairobi having lower coexistence, and Makueni/Bomet *C. hemipterus* showing dominance. Across Africa, *C. hemipterus* prevails, especially in central and coastal areas, while *C. lectularius* has lower suitability, with isolated high-suitability zones. Both species coexist in central/southern Africa, parts of the east, and coastal areas in septentrional/west Africa. The Sahara’s extreme conditions challenge both bed bug species’ survival, emphasizing climate’s role in their infestation and distribution dynamics. Insights into bed bug ecology in Africa underscore the need for comprehensive pest management and public health strategies in the continent.

## Simple Summary

This study explores the dynamics of bed bug infestations in Africa, focusing on two species: the common (*Cimex lectularius*) and the tropical (*Cimex hemipterus*) bed bugs. These bugs, known for feeding on blood, are not only a nuisance but can potentially transmit diseases. The research utilized system dynamics modeling for infestation and ecological niche modeling to analyze their potential distribution across different climates and habitats and their host availabilities in Africa. The findings revealed varied infestation patterns in Kenya, with a high prevalence of *C. lectularius* in Mombasa and dominance of *C. hemipterus* in regions like Makueni and Bomet. This study maps the potential spread of these species across Africa, highlighting areas with high coexistence risks. This research is crucial as a prerequisite for developing effective pest management strategies and public health measures to combat bed bug infestations, thus safeguarding local and regional food supplies and overall human health.

## 1. Introduction

Bed bugs (Hemiptera: Cimicidae) are small, blood-feeding insects that have adapted to live in close association with humans and other warm-blooded animals [1]. They are notorious nocturnal nuisances that have plagued human dwellings for centuries and have resurged globally over the past two decades, garnering renewed attention from both the scientific community and the public [2,3]. They are not only a source of physical discomfort but also potential vectors of various pathogens, making their study critical for public health and pest management efforts [4]. Bed bugs are not only a household pest but also a public health concern [5]. The bugs have been associated with insomnia, social stigma, anxiety, restlessness, stress, nightmares, and depression [6,7]. Furthermore, their bites are attributed to skin irritation with severe itching, and cutaneous manifestations such as pruritus, papules, and erythema [8]. Bed bug feces contain high amounts of histamine, which is responsible for itching, muscle swelling, and other allergic reactions [9]. Past studies have documented high costs of bed bug management, with an average cost of USD 463–482 per apartment, while whole-house pest management can be USD 2000–4000 [10]. Additionally, the resurgence of bed bugs in the wake of increased international travel frequencies and pesticide resistance underlines the urgency of comprehensive research in this area.

Within the Cimicidae, there are several species, but two of the most well-known and widely distributed species are *Cimex lectularius* L. (common bed bug) and *Cimex hemipterus* (tropical bed bug) F. (Hemiptera: Cimicidae) [11,12]. These two species exhibit distinct characteristics and have varying global distributions. Historically, *C. lectularius* was found worldwide, particularly in temperate regions, infesting human dwellings and occasionally poultry houses [13,14]. However, following widespread pesticide use in the mid-20th century, *C. lectularius* populations declined significantly in many areas, though currently there is resurgence of this pest in various parts of the world [11]. This resurgence is attributed to factors such as increased international travel, pesticide resistance, and reduced public awareness. The pest primarily infests human habitats, including homes, hotels, hostels, and even public transport systems. On the other hand, *C. hemipterus* is commonly found in tropical and subtropical regions, particularly in Africa, Asia, and some parts of the Americas [15], and is less prevalent in temperate climates. Unlike *C. lectularius*, which primarily infests human habitations, *C. hemipterus* has a broader habitat range including human dwellings, as well as bat colonies and bird nests [11,16]. While *C. lectularius* and *C. hemipterus* are the most known bed bug species, there are other species within the Cimicidae with more localized distributions. These include species like *Cimex pilosellus* (Horváth) and *Cimex adjunctus* Barber (Hemiptera: Cimicidae), which are often associated with specific hosts or habitats [17,18]. Recent studies have documented the presence and distribution of *Cimex* species across various regions in Africa. A study by Deku et al. [15] assessed *C. hemipterus* infestations in Cape Coast, Ghana, highlighting the prevalence of this species in West Africa. In Kenya, research by Mbuta et al. [1] examined household perceptions and infestation dynamics of bed bugs, providing insights into the widespread occurrence of *Cimex* species in East Africa. Additionally, Fourie and Crafford [19] discussed the resurgence of bed bugs in Africa, noting increased reports of *C. hemipterus* in countries such as Tanzania, Senegal, Nigeria, Gambia, Kenya, Sierra Leone, Zimbabwe, and Ghana.

The distribution of bed bug species is influenced by various factors, including climate, host availability, human movement, and adaptations to different environments [16]. The resurgence of bed bugs in recent years has led to renewed interest in their biology, ecology, behavior, and distribution [17]. Understanding the diversity and distributions of common and tropical bed bugs is crucial for effective pest management and public health efforts, especially in regions where bed bugs are a growing concern. In the African context, where diverse ecosystems and climatic conditions create a unique ecological landscape, understanding the distribution and overlap of bed bug species is paramount for their management [15,16]. While many studies have explored the biology and behavior of bed bugs, there is a notable gap in research focused on the African continent, which is home to a rich diversity of habitats and climates [16,17]. Our study therefore aims to bridge the knowledge gap by delving into the analysis of potential distribution patterns and the intriguing phenomenon of two distinct bed bug species (*C. lectularius* and *C. hemipterus*) co-infesting in African environments. With a focus on *C. hemipterus* and *C. lectularius*, the most prominent bed bug species worldwide, we seek to elucidate their infestation dynamics, ecological niches, and interactions in Africa.

Africa’s diverse landscapes, ranging from arid deserts to lush rainforests, provide a unique setting to study bed bug ecology. A combination of system dynamics modeling and ecological niche modeling techniques was employed to investigate the infestation dynamics and potential distribution of *C. hemipterus* and *C. lectularius* across the continent. Specifically, this study aimed to (i) analyze the infestation dynamics of *C. hemipterus* and *C. lectularius* in Kenya by evaluating their prevalence and influencing factors in different locations through interview data collection and system dynamics modeling, (ii) assess the current distribution of *C. hemipterus* and *C. lectularius* across Africa by conducting field sampling and identification of specimens collected from various locations, and (iii) predict the potential habitat suitability of the two species and their co-occurrence across the African continent using ecological niche modeling, incorporating key environmental variables such as temperature, humidity, and human population density. By shedding light on the distribution and overlapping of these two bed bug species in Africa, our research aims to not only enhance our understanding of bed bug ecology in the continent but to also serve as a valuable reference for researchers, policymakers, and pest control professionals working toward sustainably mitigating the impact of these resilient and enigmatic insect pests.

## 2. Materials and Methods

### 2.1. Evaluation of C. hemipterus and C. lectularius Infestation Dynamics in Different Counties in Kenya

#### 2.1.1. Interview and Data Collection for Infestation Dynamics

Face-to-face interviews were conducted with a total of 400 randomly selected respondents, 100 from each of the four counties in Kenya (Nairobi, Mombasa, Makueni, and Bomet). The interviews were conducted using a semi-structured questionnaire prepared in English (Table S1). To avoid bias (information and variables) and improve the understanding between the enumerator and the respondent, the interviewers translated the questionnaire into the local native language or Kiswahili. Household heads willing and who consented to participate were included as respondents in the study. Additionally, in the absence or inability of the household head to participate in the interview, an adult from the household was interviewed instead. The information collected included knowledge and perception of bed bug presence.

#### 2.1.2. Infestation Dynamics Model Development

The approach involved employing the infestation dynamics model for the two species of bed bugs, grounded in the principles of systems thinking and its associated archetypes, including Causal Loop Diagrams (CLDs), Reinforcing (R), and Balancing (B) loops ([1]). Although these archetypes are qualitative in nature, they prove indispensable in revealing the fundamental feedback mechanisms that come into play in households and their surrounding environments when grappling with pest infestations such as bed bugs. To create a dynamics model, we translated the CLD into a set of interconnected elements, encompassing stocks, flows, auxiliary links, and clouds. Subsequently, these components were transformed into a system of coupled differential equations, Equations (1)–(3), to facilitate simulations [20].

(1) $$\frac{dS}{dt}=\frac{\beta }{N}SI+\gamma I+\alpha T\;$$

(2) $$\frac{dI}{dt}=\frac{\beta }{N}SI-(\gamma +\tau )I\;$$

(3) $$\frac{dT}{dt}=\tau I-\alpha T\;$$

where *β* > 0, *τ* > 0, *α* ≥ 0, and *γ* > 0. The total population size is *N* = *S*(*t*) + *I*(*t*) + *t*(*t*). The initial conditions are satisfied when *S*(0) > 0, *I*(0) > 0, *T*(0) ≥ 0 and *S*(0) + *I*(0) = *N*, where N is the constant total population size, and *dN*/*dt* = 0.

**Figure 1:**
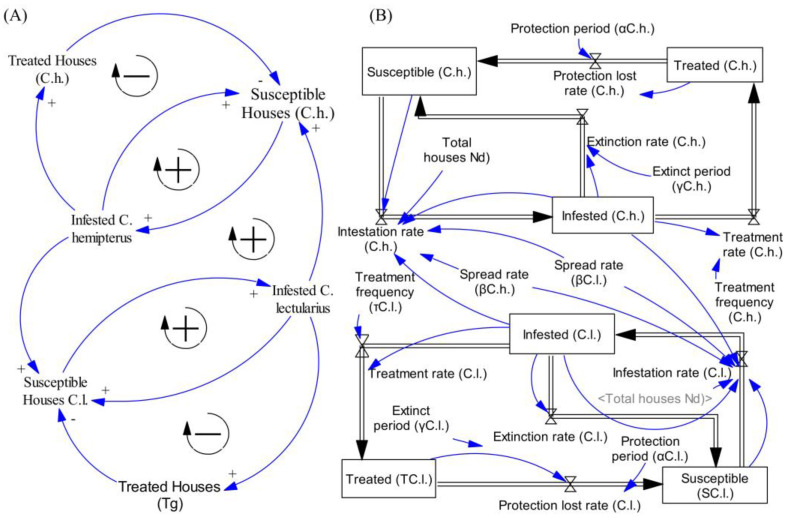
Susceptible–Infested–Treatment (SIT) model translated into Causal Loop Diagram (**A**) and stock and flow diagram (**B**) for infestation dynamics of *Cimex hemipterus* (C.h.) and *Cimex lectularius* (C.l). The arrows indicate cause-and-effect relationships. Positive arrows mean a direct proportional relationship between cause-and-effect, while negative arrows indicate an inverse proportionality.

In the case of the Susceptible–Infested–Treatment (SIT) model, a Causal Loop Diagram was crafted to illustrate cause-and-effect relationships. In this diagram, positive arrows signify a direct proportional relationship between cause and effect, while negative arrows indicate an inverse proportionality in situations where both bed bug species coexist. Within this CLD (Figure 1A), each species’ infestation dynamics is influenced by two feedback loops: (1) A positive loop: as the number of infested houses increases, the likelihood of other houses becoming susceptible to infestation also rises, resulting in a higher count of infested houses. (2) A negative loop: as the number of infested houses grows, the number of treated houses also increases, leading to a reduction in the count of susceptible houses. Additionally, a fifth loop arises from the interaction between the two bed bug species, where a house infested with *C. hemipterus* becomes susceptible to *C. lectularius*, and vice versa. The Causal Loop Diagram (CLD) is presented in Figure 1A, while Figure 1B displays the stock and flow diagram, along with auxiliary variables derived from the CLD.

The model utilizes “susceptible”, “infested”, and “treated” houses as stocks within the system, representing the number of houses that are susceptible, infested, and treated at any given point in time. The rates of change are depicted through the in-flow and out-flow connections in the diagram. Auxiliary components and constants that influence the system’s behavior are interconnected using information arrows, linking them to flows and stocks to represent the relationships among variables in the form of equations. The stock and flow diagrams for each of the two bed bug species exhibit interconnections between the two species (Figure 1B).

The survey data on prevalence, knowledge, perceptions, and self-reporting, in addition to the respondents’ reported control mechanisms and their average time of effectiveness (Table S2), were used for model setting and simulations. All the model commodities and units were checked before performing the simulations. Simulation and implementation of the models were carried out using the Vensim PLP 8.1 platform (Ventana systems, Harvard, Cambridge, MA, USA). This platform consists of a graphical environment that usually permits the drawing of a Causal Loop Diagram (CLD) and a stocks and flow diagrams and enables one to carry out simulations.

### 2.2. Prediction of the Potential Distribution of C. hemipterus and C. lectularius and Their Co-Occurrence Across the African Continent

#### 2.2.1. Sample Collection for Potential Distribution

Two types of data were collected, primary and secondary, across different regions in Africa based on specific criteria. First, countries were chosen to represent diverse climatic zones (tropical, subtropical, and arid) to assess the ecological adaptability of the two Cimex species. Second, priority was given to countries with documented reports of infestations from published research, government health records, or pest control agencies. Additionally, sampling focused on regions with varying human population densities and urbanization levels, as bed bugs are closely associated with human habitats. Countries with major trade and travel hubs (such as international airports, seaports, and border crossings) were included to evaluate the role of human movement in the spread of *Cimex* species. To ensure a continent-wide perspective, collection sites were geographically distributed across East, West, Central, and Southern Africa, providing a balanced representation of different ecological and socio-economic contexts. Lastly, feasibility and logistics were considered, including accessibility to study sites, availability of local collaborators, and required permits for specimen collection. Therefore, specimens were sampled as primary data in Ghana, Kenya, and Togo. The samples were collected through institutional collaborations involving partnerships with local health departments, research institutions, and community stakeholders. Study permits such as the National Commission for Science, Technology & Innovation—License No: NACOSTI/P/20/7147 and the Institutional Certificate of Ethical Clearence—ICIPE/IBC/02/2020 were obtained. Live bed bug samples were disturbed and flushed from their hiding places (wall crevices, cracks, bed frames, cupboards, and mattresses) using a pair of forceps. Once collected, bed bugs were preserved in absolute ethanol in 50 mL falcon tubes (well-marked indicating each reference collection point), then transported to the *icipe* Arthropod Pathology Unit and stored at −20 °C awaiting molecular analysis. Samples were identified using molecular tools through amplification and sequencing part of the Cytochrome Oxidase subunit I (CO1) gene as *C. hemipterus* and *C. lectularius* (Mbuta et al., unpublished data). The geographical coordinate associated with each sampling point was recorded and the dataset compiled was then mapped to assess the distribution across Africa. Furthermore, secondary data (geographical coordinate of reported points of each species) were extracted from the literature on other reported infested countries such as Mali, Burkina Faso, Benin, Cameroon, Angola, Uganda, Tanzania, Zambia, Malawi, Zimbabwe, Mozambique, and Madagascar.

#### 2.2.2. Environmental Data

The environmental variables used were obtained from the Global Climate Data official website, Worldclim (http://www.worldclim.org/bioclim.htm (accessed on 19 October 2023)) [21], and the Global Land Cover Facility (GLCF). These variables comprise 19 bioclimatic factors, land cover, and elevation data. Minimizing the collinearity between predictors was imperative; and this was achieved by performing a collinearity test on all variables by filtering them following a similar approach to that documented by Mbuta et al. [1] and Wang et al. [22]. The MaxEnt model was run first using the distribution data of each bed bug species and the bioclimatic factors in order to obtain the percentage contribution of each variable. All of the distribution points were then imported in Arc-GIS, and the attribute variables of the bioclimatic variables were extracted. Additionally, the “virtual species” package [23] in R-software, version 4.2 (R Foundation for Statistical Computing, Vienna, Australia) was used to explore the extracted variables’ clusters spatial correlation using Pearson’s correlation coefficient [24]. Finally, temperature and relative humidity were the two environmental predictor variables that were used to establish the potential geographical distribution of the two bed bug species.

#### 2.2.3. Ecological Niche Model Development Calibration

The two key environmental predictor variables previously established and reported in the literature (temperature and relative humidity) [25,26] and the host (human population) were used for ecological niche model development. Mean annual temperature was sourced from the WorldClim platform (www.worldclim.org (accessed on 19 October 2023)) at approximately 5 km spatial resolution [27,28], while relative humidity was retrieved from the CliMond historical dataset interpolated at 10 km spatial resolution (available at https://www.climond.org (accessed on 27 October 2023)) [29] (available at https://www.climond.org (accessed on 27 October 2023)) [29]. To represent the host (human population) density projection for 2020 in the selected areas, the data were sourced from WorldPop (https://www.worldpop.org/ (accessed on 30 October 2023)) as the number of people per pixel (pixel size was 1 × 1 km) [30].

Species occurrence data were utilized together with the environmental variables and population density to develop habitat suitability predictive models using the ecological niche approach. The modeling experiment was conducted using the maximum entropy (MaxEnt, version 3.4) algorithm, a machine learning technique that utilizes the principle of maximum entropy to estimate the distribution or probability of occurrence [31]. This modeling approach was considered due to its statistical robustness, adaptability to various environments, relatively small sample size requirement, and presence-only data [32]. The models were replicated three times using the sub-sample method, and an ensemble of the three probability outputs was considered to determine the optimum suitability and performance of the models. Seventy percent (70%) of the respective species occurrence points were used for training, while 30% were retained for testing model performance. The model outputs, which are suitability scores ranging from 0 (very low) to 1 (optimal), were mapped using QGIS 3.10.9 software (https://qgis.org/ (accessed on 25 March 2022)).

To validate the robustness of the model, a threshold-independent area under the receiver operating characteristics (ROC) curve (AUC) analysis was used. The AUC values ranged from 0.5 (lowest predictive ability) to 1 (highest predictive ability) [1,33]. Therefore, the excellently performing model was selected to predict the distribution of *C. hemipterus* and *C. lectularius* bed bug species in Africa.

#### 2.2.4. Co-Suitability Habitat of *Cimex hemipterus* and *Cimex lectularius*

To identify biogeographical regions overlapping of *C. hemipterus* and *C. lectularius* suitability areas, we used the specialized probabilistic techniques, dubbed the Jaccard index [34,35,36,37]. The Jaccard index is defined as the ratio of the size of the intersection over the union for any two finite sets, U and V, as given in the equation below [38].

(4) $$f\left(U,V\right)=\frac{\left|U\cap V\right|}{\left|U\cup V\right|}$$

Its value ranges from 0 to 1, i.e., 0 ≤ *f* (*U*, *V*) ≤ 1. The greater the value of *f* (*U*, *V*), the more similar the sample sets are. Its interpretation is simple and straightforward; however, it is more accurate for binary data [39]. The index was implemented using R statistical software [24].

## 3. Results

### 3.1. The Infestation Dynamics of C. hemipterus and C. lectularius in Different Counties of Kenya

**Figure 2:**
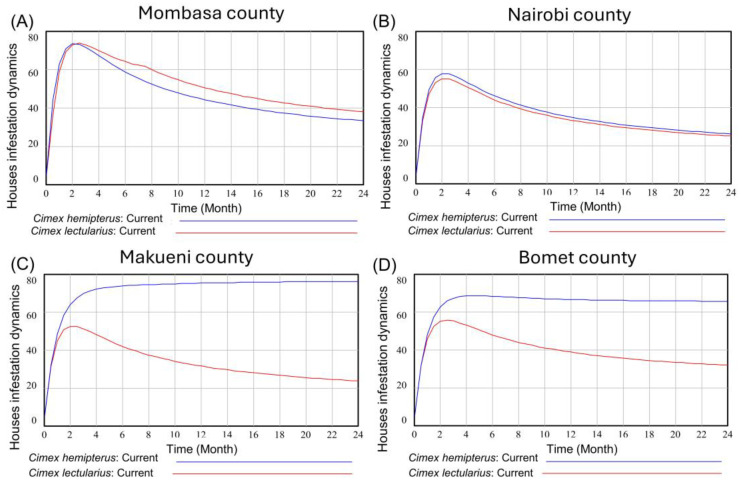
Infestation dynamics of *Cimex hempterus* and *Cimex lectularius* in houses in Mombasa (**A**), Nairobi (**B**), Makueni (**C**), and Bomet (**D**) counties in Kenya.

While both species of bed bugs were found across the counties, the infestation dynamics of these two species exhibited distinct patterns. Notably, Mombasa (Figure 2A) experienced higher levels of infestation, with *C. lectularius* prevailing over *C. hemipterus* after 4 months. In Nairobi County (Figure 2B), the two species coexisted, but the overall infestation levels were lower than infestations observed in Mombasa. Furthermore, in Makueni (Figure 2C) and Bomet (Figure 2D) counties, the infestation dynamics displayed distinctive characteristics. Specifically, *C. hemipterus* infestations were considerably higher and dominated over *C. lectularius*, albeit at a lower intensity.

### 3.2. Current Distribution of C. hemipterus and C. lectularius

**Figure 3:**
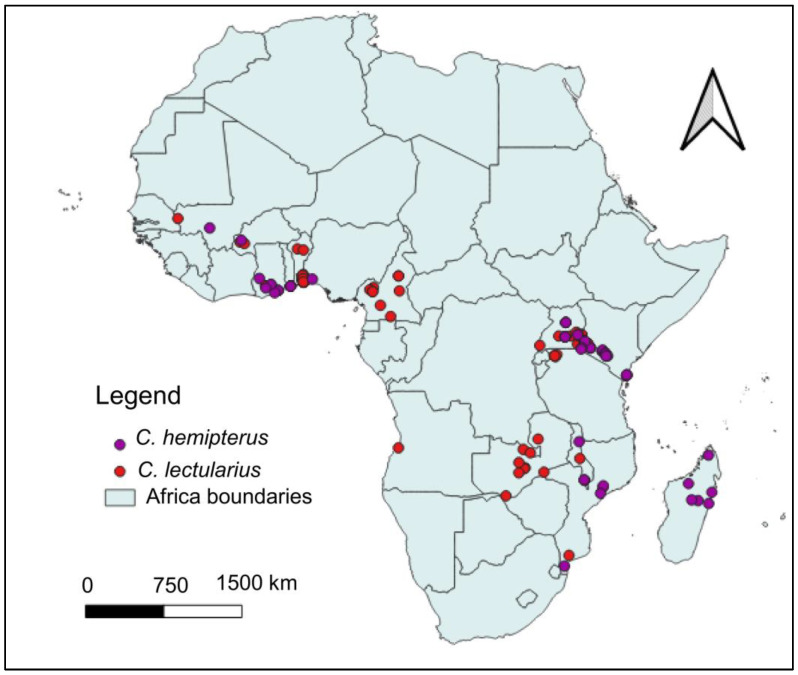
Geographical distribution of *Cimex hemipterus* and *Cimex lectularius* across Africa based on field sampling.

The geographical distribution of *C. hemipterus* and *C. lectularius* across Africa based on field sampling data is illustrated in Figure 3. The distribution points highlight regions where these species were recorded, revealing distinct and overlapping occurrence patterns. *Cimex hemipterus* is widely distributed in West, East, Central, and Southern Africa, with notable occurrences in Ghana, Senegal, Kenya, Tanzania, Madagascar, and Southern African countries. Similarly, *C. lectularius* showed a broader presence in West, East, and Southern Africa, with significant records in Kenya, Tanzania, Ethiopia, and several West African countries. Both species co-occur in West and East Africa, particularly in Ghana, Senegal, Kenya, and Tanzania, while *C. hemipterus* appears dominant in Madagascar.

### 3.3. Potential Habitat Suitability of C. hemipterus and C. lectularius Bed Bug Species in Africa

**Figure 4:**
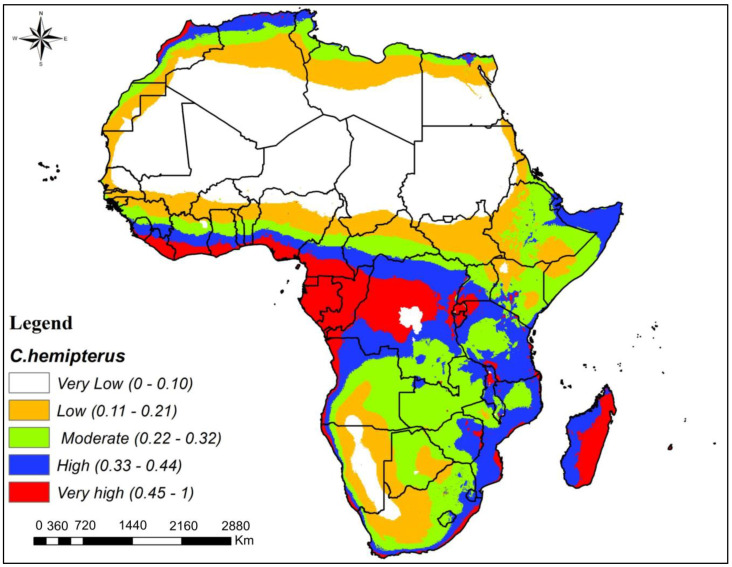
Potential geographical distribution of *Cimex hemipterus* in Africa.

The output map for *C. hemipterus* (tropical bed bug) distribution in Africa under current climate (Figure 4) shows that most African regions were moderate- to very high-suitability zones. The eastern and central regions of Africa were predicted as high and very high, while other regions ranged from low to moderately suitable zones (Figure 4). Very low-suitability zones were found mainly in the northern and Sahel regions, though they could be scantly distributed across other parts. Central and eastern regions of Africa, including areas in countries like Tanzania, Eritrea, and Uganda, were predicted to have high to very high suitability for *C. hemipterus*. These regions offer conducive/favorable conditions to the survival and proliferation of this bed bug species. Other regions across Africa displayed a range of suitability levels, from low to moderate (Figure 4).

**Figure 5:**
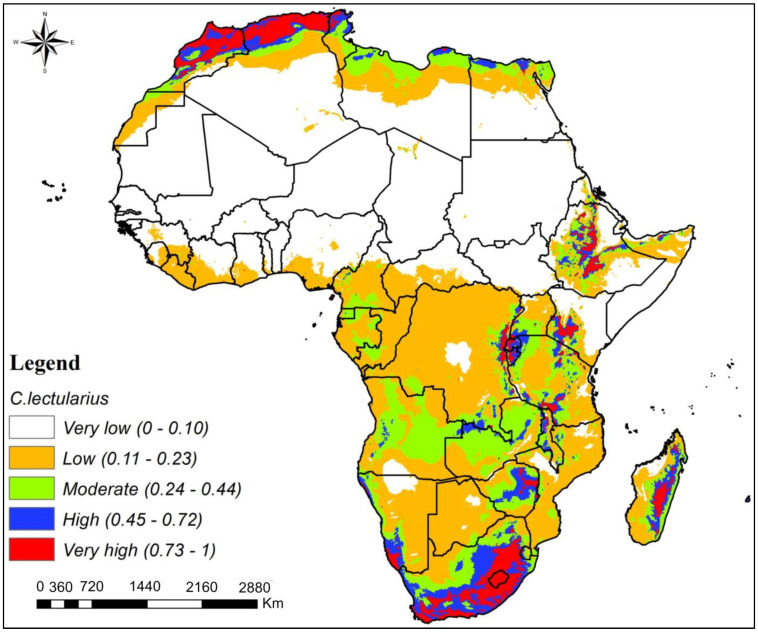
Potential geographical distribution of *Cimex lectularius* in Africa.

Across Africa, the output map of *C. lectularius* species distribution shows that most of the regions comprising northern, central, and southern parts ranged from low- to very low-suitability zones (Figure 5). However, the far northern and southern regions of Africa ranged from highly to very highly suitable zones for *C. lectularius* survival and proliferation, although these regions were also fragmented across several other parts of the continent (Figure 5).

### 3.4. Co-Suitability Habitat of C. hemipterus and C. lectularius in Africa

**Figure 6:**
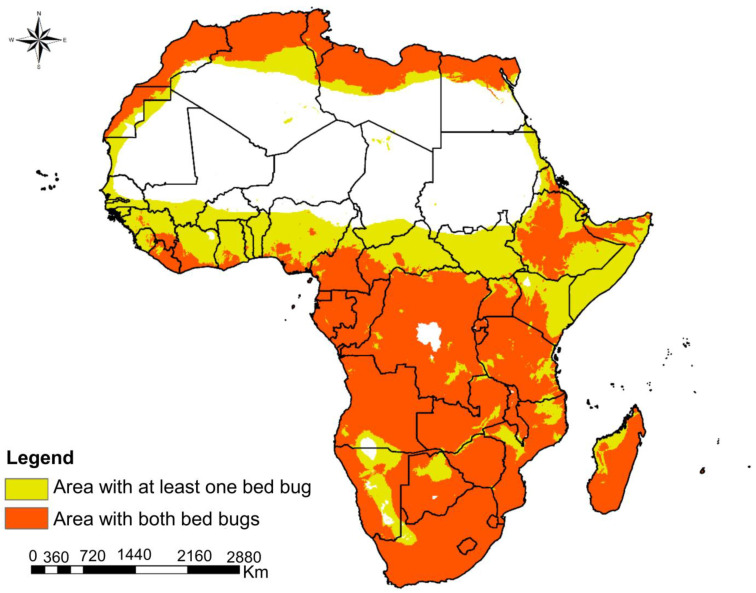
Niche overlap of two bed bug species, *Cimex hemipterus* and *Cimex lectularius,* in Africa.

The two species potentially overlap in central and southern regions of Africa and most parts of the eastern region (Figure 6). The co-suitable habitat was also found at coastal parts of septentrional and West Africa.

### 3.5. Cimex hemipterus and C. lectularius Bed Bug Species Habitat Suitability Model

**Figure 7:**
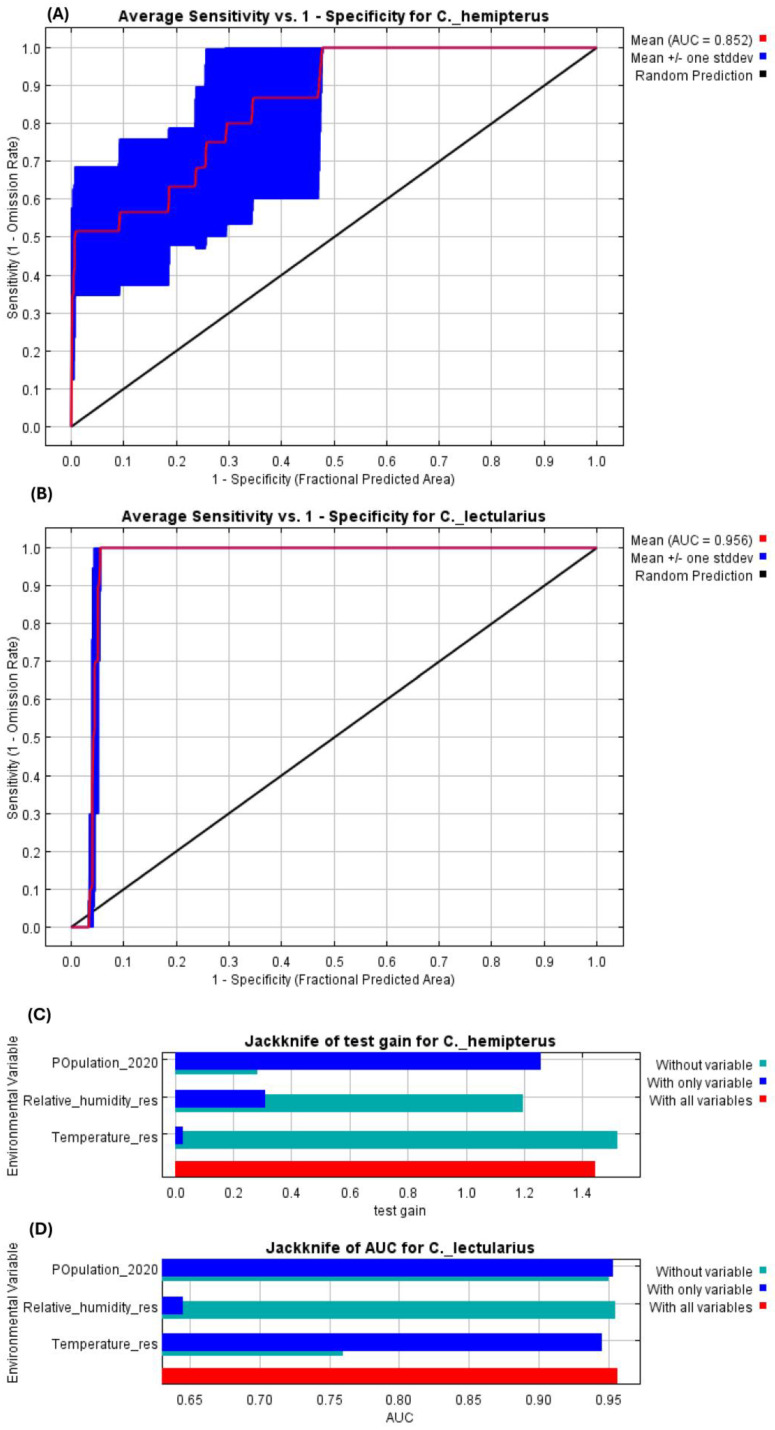
Receiver operating characteristic (ROC) curve verification of the predicted potential habitat for the two bed bug species in Africa: *Cimex hemipterus* (**A**) and *Cimex lectularius* (**B**). The relative importance of environmental variables for predicting the habitat suitability of *Cimex hemipterus* (**C**) and *Cimex lectularius* (**D**) in Africa.

The suitability model for both species recorded AUC values greater than 0.8, suggesting a moderate occurrence area of the insect pest species (Figure 7A,B). This indicates that our model successfully predicted the habitat suitability of the two bed bug species *C. hemipterus* and *C. lectularius* in Africa.

The model jack-knife test showed that the environmental variables, namely, population, relative humidity, and temperature, are the most important variables in determining the suitability of the two bed bug species in Africa (Figure 7C,D).

## 4. Discussion

In Kenya, the infestation dynamics analysis of *C. hemipterus* and *C. lectularius* revealed a dominance infestation of *C. lectularius* in Mombasa and an overlap of the two species in Nairobi, while the native species *C. hemipterus* dominates in Makueni and Bomet counties. Several factors could have contributed to this phenomenon. As a primary gateway for imports, Mombasa, a coastal region city with a major port, deals with a substantial flow of goods, including cargo in shipping containers. Bed bugs, known for their ability to hide and travel within various objects, could easily hitch a ride in these shipments. Infested containers, textiles, or furniture can introduce the invasive *C. lectularius* to the region [4]. Mombasa’s role as a port city and a popular travel destination leads to a constant influx of people. Tourists, travelers, and migrant workers often bring bed bugs with them inadvertently, facilitating the establishment of this pest in the local environment [40]. The bustling economic activity of Mombasa, largely centered around shipping and tourism, also attracts diverse population groups, leading to a high density of accommodation facilities, both formal and informal. This concentrated human housing increases the likelihood of bed bug infestations [41]. In such a setting, *C. lectularius*, known for its adaptability to urban environments, could also outcompete native species.

Nairobi, the capital city of Kenya, is characterized by its status of being a major tourism hub and its busy international airport. In this urban cosmopolitan environment, both *C. lectularius* and the native *C. hemipterus* coexist, reflecting a unique set of circumstances. Nairobi has a reputation as a tourist destination and a crossroad of several connections due to its international airport with a global migration network of travelers. Tourists and business travelers frequently visit Nairobi and could potentially introduce invasive bed bug species from various parts of the world in addition to the native species [40]. The constant flow of people in and out of the city, including domestic and international travelers, could also lead to the inadvertent introduction and distribution/spread of bed bugs [40]. The co-occurrence of *C. lectularius* and *C. hemipterus* may result from a balance between the species’ adaptability and competition for resources. Nairobi’s bustling economy supports diverse commercial activities, including the trade of used furniture and clothing. Such exchanges can easily facilitate the spread of both bed bug species, as they readily infest these items [1]. In contrast to the coastal and urban regions, Makueni and Bomet exhibit the dominance of the native species, *C. hemipterus*. These regions generally experience fewer international travelers and less trade activity. Consequently, the introduction of invasive species like *C. lectularius* in the two counties is less likely. In addition, the economic activities in Makueni and Bomet may be less diverse and more agriculture-centric. Such regions may have lower population densities and less urbanization, reducing the spread of bed bugs [42]. In addition, *C. hemipterus*, as a native species, may be better adapted to the local environment and climatic conditions of these areas, giving it a competitive advantage.

The potential distribution of bed bug species across Africa is primarily low, particularly in regions corresponding to the Sahara Desert. The extreme climatic conditions, characterized by scorching temperatures exceeding 104 °F (40 °C) [43,44] and low relative humidity dropping below 10% [45,46], create an inhospitable environment for bed bugs, especially *C. lectularius* and *C. hemipterus* [47]. The harsh desert conditions, including aridity and frequent temperature fluctuations, may contribute to low bed bug survival. The extreme heat poses a significant challenge to bed bugs [26], as ectothermic insects, whose body temperature is optimally regulated by the external environment [25,48]. Prolonged exposure to high temperatures can lead to desiccation, causing rapid water loss in the insect due to poor adaptability of its exoskeletons to retain moisture in arid conditions. Additionally, the Sahara’s temperature extremes may disrupt the bed bug’s reproductive cycle, affecting egg laying and hatchability, ultimately limiting population growth. Bed bugs rely on humans or warm-blooded animals as hosts for blood meals [1], and the Sahara’s vast expanses of uninhabited desert with low population density restrict their access to suitable hosts, making it challenging for bed bug populations to establish and thrive. While the Sahara is predominantly hot and arid, occasional periods of increased humidity and milder temperatures associated with rare rainfall may provide temporary relief for bed bugs, allowing them to feed and reproduce. However, these conditions are transient and offer only short-lived respite from the prevailing arid environment.

*Cimex hemipterus* shows a distinct potential distribution pattern across various regions of Africa, excluding the Sahara and its nearby environs, with a prevalence of moderate to very high suitability. This variability is attributed to differences in local climate, host availability, and environmental factors [49]. Central Africa and coastal areas emerged as highly suitable zones for *C. hemipterus* due to their tropical climate, providing optimal temperatures and humidity levels for the species’ survival and reproduction [11,50]. The species’ ecological resilience enables it to adapt to diverse habitats, contributing to its widespread distribution within suitable regions.

The potential distribution of *C. lectularius* across Africa reveals a pattern of low to very low suitability zones in most parts of the continent, with high to very highly suitable zones occurring in fragmented pockets. These patterns suggest generally unfavorable conditions for *C. lectularius* in northern, central, and southern Africa [1,10]. In contrast to *C. hemipterus*, *C. lectularius* primarily infests human habitations, including homes, hotels, hostels, and public transportation [50,51]. Historically, *C. lectularius* had a global distribution, but factors like human activity, trade routes [52], and the use of pesticides, especially DDT [8], have led to a decline in its populations. However, recent years have seen a resurgence of the pest in various parts of the world, including some regions of Africa, which is attributed to increased international travel, pesticide resistance, and reduced public awareness [1,17].

The potential co-occurrence of *C. hemipterus* and *C. lectularius* in central and southern regions of Africa, along with significant parts of the eastern region and coastal areas in septentrional and West Africa, indicates favorable conditions for both species. These regions exhibit moderate to high suitability for both bed bug species, suggesting that factors such as climate, habitat availability, and host populations support their simultaneous thriving [25,53,54]. Coastal regions, including areas along the Mediterranean Sea and the Atlantic Ocean, demonstrate co-suitable habitats for both bed bug species. Coastal climates with moderate temperature and humidity levels may facilitate high reproductive rates of bed bug populations, and milder conditions compared to inland areas can favor their survival.

Several factors contribute to the potential co-occurrence of these bed bug species in these regions. Adequate nesting sites in human dwellings, along with the presence of suitable hosts like humans and animals, play crucial roles in supporting bed bug populations [1,12]. Human movement, trade, and tourism may contribute to the spread and potential co-occurrence of both species. However, further research is needed to delve into specific factors driving their co-occurrence and provide insights into their ecology, behavior, and interactions. The dominance of high-suitability zones for *C. hemipterus* in Central Africa and coastal regions implies a higher risk of infestations in these areas. Thus, pest control measures, including surveillance, education, and targeted interventions, should be prioritized to prevent, contain, and manage bed bug infestations effectively. On the other hand, the prevalence of low-suitability zones for *C. lectularius* in much of Africa suggests a lower risk of infestations in these areas. However, the presence of isolated high-suitability zones underscores the need for vigilance and proactive pest control measures. Given the co-suitable habitat for the two bed bug species in these regions, comprehensive, sustainable, and effective pest management strategies are crucial. Integrated pest management practices, public awareness campaigns, and vigilant monitoring are essential components to address and prevent bed bug infestations effectively.

## 5. Conclusions

Bed bug species infestation in Kenya may be shaped by factors such as international trade and travel, with invasive *C. lectularius* thriving in regions with high human mobility, population density, and economic activity. Native species like *C. hemipterus* may dominate in areas with lower international travel and economic diversity. The Sahara Desert’s extreme climate, characterized by scorching temperatures and low humidity, creates a challenging environment for bed bugs. Hence, bed bugs face limitations in survival and reproduction in such conditions. However, localized factors like human activities, urbanization, and suitable hosts, contribute to the persistence of bed bug populations in specific spots within or near the Sahara. The distribution pattern of *C. hemipterus* in Africa involves a complex interplay of climate, environmental factors, and human activity. Central Africa and coastal areas exhibit high suitability, while arid regions and the Sahara present harsh conditions that are less suitable. Understanding these patterns is crucial to developing effective pest management strategies and public health measures. *Cimex lectularius* distribution is influenced by climate and human activities, with most parts of Africa characterized by low suitability, while high-suitability zones occur in fragmented pockets. The co-suitable habitat for both species in various African regions emphasizes the need to unravel the mechanisms underlying their coexistence for successful pest control and public health initiatives.

## Data Availability

The raw data supporting the conclusions of this article will be made available by the corresponding author upon reasonable request.

## Supplementary Materials

The following supporting information can be downloaded at https://www.mdpi.com/article/10.3390/insects16040395/s1: Table S1: Questionnaire modules for the assessment of bed bugs management and control practices among residents in different counties in Kenya; Table S2: Parameters for models’ simulations.
