# Supplementary material for: Know Where You Go: Infestation Dynamics and Potential Distribution of Two Bed Bug Species (Hemiptera: Cimicidae) in Africa
Source: Insects. 2025 Apr 9;16(4):395. doi: 10.3390/insects16040395 (PMC12027950; doi:10.3390/insects16040395)
Supplement: Supplementary file 1 [file insects-16-00395-s001.zip › insects-3503837-supplementary.pdf]

Appendix

Table S1: QUESTIONNAIRE MODULES FOR THE ASSESSMENT OF BED BUGS MANAGEMENT AND CONTROL PRACTICES AMONG RESIDENTS IN DIFFERENT COUNTIES IN KENYA.

Introductory statement:

"Dear Sir/madam, I work for the International Centre for Insect Physiology and Ecology (*icipe*). We are conducting a survey to study the level of infestations, economic impact, perceptions, knowledge, public awareness and practices regarding bed bugs control and management around the country. Your response to these questions would remain anonymous. Taking part in this study is voluntary. We request that you answer the questions as accurately and honestly as possible so that our understanding and future activities are then based on addressing the real pest control, management and problems faced by residents like yourself. If you choose not to take part, you have the right not to participate and there will be no sequences. Thank you for your kind co-operation."

MODULE 1: HOUSEHOLD AND VILLAGE IDENTIFICATION

**1.1 Household Identification**	**Code**
**1**. County	
**2**. Sub-county	
**3**. Ward	
**4**. Location	
**5**. Village:	
**6**. Age of respondent	
**7**. Name of household head (three names):	
**8**. Sex of household head 1=Male 0=Female	
**9**. Name of the respondent (three names):	
**10**. Sex of respondent 1=Male 0=Female	
**11**. Name of respondent's spouse	
**12**. Cell phone number of household head	

**1.2 Interview details**	**Code**
**14**. Date of interview (dd/mm/yyyy):	/ / 2020
**15**. Time started (24 HR)	
**16**. Name of enumerator	
**17**. Name of supervisor:	
**18**. Name of data entry clerk	

**GPS reading of homestead**	
**19**. Way point number	
**20**. Latitude (North)	
**21**. Longitude (East)	

**13.** Cell phone number of the spouse:													**22.** Altitude (meter above sea level)	

MODULE 2: HOUSEHOLD COMPOSITION, CHARACTERISTICS AND HOUSING CONDITIONS

2.1 HOUSEHOLD COMPOSITION AND CHARACTERISTICS (*Household members: persons who live together and eat together from the same pot (share food), including hired labour, students and spouse living and working in another location but excluding visitors*)

ID CODE	Name of household member [Start with respondent]	Sex 1=M 0=F	Relationship to the household head **CODE 1**	Age (complete years; 0 if less than 1 year)	Marital status? **CODE 2**	Education (years) **CODE 3**	Primary occupation **CODE 4**	How many months in the past year was [NAME] present in the household?	Number of children living in the same household (under age of 18yrs)	Number of children living in the same household (above age of 18yrs)
	**AA1**	**AA2**	**AA3**	**AA4**	**AA5**	**AA6**	**AA7**	**AA8**		
1										
2										
3										
4										

**CODE 1**	**CODE 2**	**CODE 3**	**CODE 4**
1.Household head<br>2.Spouse<br>3.Son/daughter<br>4.Parent<br>5.Son/daughter-in-law<br>6. Grandson/granddaughter<br>7.Other relative<br>8.Hired worker<br>9.Other, specify……………	1.Married living with spouse<br>2.Married living without spouse<br>3.Divorced/separated<br>4.Widow/widower<br>5.Never married	0. None/Illiterate<br>1. Adult education or 1 year of education<br>* Give other education in years (e.g. 2 yrs for std 2, 8 yrs for class 8 etc)<br>100. Religious education	1.Farming (crop+ livestock)<br>2.Salaried employment<br>3.Self-employed off-farm<br>4.Casual labourer on-farm<br>5.Casual labourer off-farm<br>6.School/college child<br>7.Non-school child<br>8.Other, specify…………..

MODULE 3: BED BUG CONTROL AND MANAGEMENT PRACTICES

3.1. Have you experience bed bugs menace/nuisance before?

1. Yes( )
2. No( )

3.2. If yes, for how long have you encountered bed bugs?

1. 1-5 years ( )
2. 6-10 years ( )
3. 10-15years ( )
4. 16-20 years ( )
5. Above 20years ( )

3.3. What are some of the methods you have tried to manage them?

1. Burn with hot water ( )
2. Exposing infested items to sunlight outside ( )
3. Use of pesticides ( )
4. Any other specify ( )

3.4. Which of the above-mentioned methods has been the most effective?

3.5. On the above-mentioned effective method(s), was it a one-time treatment or on repeated occasions?

1. Once ( )
2. Repeated ( )

3.6. How many pesticides have you tried so far to eradicate the bugs?

3.7. On the above-mentioned types, which one would you recommend as the most effective pesticide?

1. None ( )
2. Any other specify ( )

3.8. After fumigation, how long does it take for the bugs to disappear?

1. 1 month ( )
2. 2 months ( )
3. 3 Months ( )
4. Above 4 months ( )

3.9. Have you ever relocated before because of the nuisance pest?

1. Yes ( )
2. No ( )

3.10. If YES, did it work for you?

1. Yes ( )
2. No ( )

3.11. How adverse are the effects of the bed bug bites?

1. None ( )
2. Mild ( )
3. Severe ( )

3.12. If severe, how did you manage(medication) them?

3.13. Which pesticide is commonly available and pocket friendly do you often use?

3.14. Was the pesticide effective enough?

1. Yes ( )
2. No ( )

3.15. If NOT, which measures did you take?

3.16. In the household items, where is the highest incidence of the bugs?
1. Furniture (   )
2. Mattresses/bedroom (   )
3. Cracks/Crevices (   )
4. Clothes(   )
5. Others Specify

3.17. Does the infestation affect your self-esteem and social life in general?
1. Yes (   )
2. No (   )

3.17. How do the general community around you perceive bed bug infestations?
1. Weird (   )
2. Normal (   )
3. Any other specify (   )

3.19. After what duration do you notice the bug bites?
1. Immediately (   )
2. 30mins-1hr (   )
3. Any other specify (   )

3.20. At what time of the day are the bugs most active?

1. Morning (   )
2. Midday (   )
3. Night (   )

MODULE 4: INFRASTRUCTURE

4.1 INFRASTRUCTURE (*all distances in walking minutes*)

4.1.1 Give the estimated distance to the following community infrastructure and services centers from your residences

Infrastructure	Distance (Minutes)
Village market	
Nearest source of insecticides and pesticides (dealers)	
Nearest neighboring household	
Nearest health center	

Time finished interview (24 HR) …………………….

Thank you very much for your time and participation (Please remember to thank the farmer genuinely)

The enumerator to answer section 14 below privately immediately after the interview

12.1 Did you ask questions properly? [____] 0=No 1=Yes

12.2 Overall, how did the respondent give answers to the questions [___]

1=willingly	2=reluctantly	3=with persuasion	4=it was hard to get answers

12.3 How often do you think the respondent was telling the truth [_____]?

1=rarely	2=sometimes	3=most of the times	4=all the time

Checked by Supervisor:

I (supervisor)________________________certify that I have checked the questionnaire to be sure that all the questions have been answered, and that the answers are legible.

Signed: ___________________________________ Date___/___/_____

Table S2: Parameters for models' simulations

Parameter	Definition	Value
**Bedbug species infestations**		
B_{Ch}	Infestation rate in *C. hemipterus*	0.2
Γ_{Ch}	Infested *C. hemipterus* extinction rate.	0.4
T_{Ch}	Infested *C. hemipterus* treatment rate	0.05
A_{Ch}	Protection lost rate in *C. hemipterus*	0.5
B_{Cl}	Infestation rate in *C. lectularius*	0.5
γ_{Cl}	Infested *C. lectularius* extinction rate.	0.2
τ_{Cl}	Infested *C. lectularius* treatment rate	0.1
α_{Cl}	Protection lost rate in *C. lectularius*	0.5
